# Distal radius morphometry of volar curvature along with scaphoid and lunate facet inclinations and ulnar variance in the Anatolian population

**DOI:** 10.1038/s41598-026-35123-2

**Published:** 2026-01-09

**Authors:** Pelin İsmailoğlu, Ufuk Nalbantoğlu, Okan Tok, Alp Bayramoğlu, Abdul Veli İsmailoğlu, Fatma Güler Yildirim

**Affiliations:** 1 Department of Anatomy, School of Medicine, Recep Tayyip Erdoğan University, Rize, 53020 Turkey; 2 Private Practice, Orthopaedics and Traumatology, Hand Surgery and Microsurgery, Terrace Fulya Center, İstanbul, 34394 Turkey; 3 Department of Anatomy, School of Medicine, Acıbadem Mehmet Ali Aydınlar University, İstanbul, 34752 Turkey; 4 Department of Anatomy, School of Medicine, Marmara University, İstanbul, 34854 Turkey; 5 Department of Anatomy, School of Medicine, İstanbul Beykent University, İstanbul, 34500 Turkey

**Keywords:** Distal radius fractures, Volar inclination angles, Orthopedic surgery, Anatomy, Diseases, Medical research

## Abstract

This study evaluates the morphometric characteristics of the volar distal radius, specifically volar curvature, scaphoid and lunate facet inclinations, and ulnar variance, in the Anatolian population. By identifying these population specific morphological variations, the study seeks to support improved anatomical compatibility of volar implants and enhance surgical planning for distal radius fracture fixation. Morphometric measurements were analyzed retrospectively on three-dimensional computed tomography images of 103 intact distal radii. Standardized sagittal sections were created through the midpoints of the scaphoid and lunate fossae, and volar curvature was quantified as the angle between the radial shaft axis and the volar cortex at 1 cm and 2 cm proximal to the distal radius volar rim on each section. Volar inclination angles were measured at 1 cm and 2 cm. Additionally, volar width (G), scaphoid facet inclination (SFI), lunate facet inclination (LFI), interfacet angle (IFA), and ulnar variance were evaluated. Volar curvature measured 1 cm and 2 cm proximal to the scaphoid and lunate fossae was significantly greater in males than females (*p* < .05). The mean transverse volar width was 26.5 mm, with no significant differences according to gender, age, or laterality (*p* > .05). The mean ulnar variance was − 2.0 ± 2.2 mm, while the mean LFI, SFI, and IFA were − 0.1° ± 8.4°, 26.1° ± 6.9°, and 24.2° ± 10.4°, respectively. This study demonstrates notable anatomical variations in the volar distal radius within the Anatolian population, particularly in volar curvature, scaphoid and lunate facet inclinations, and ulnar variance. These population specific morphometric differences underscore the importance of integrating multiple volar parameters into preoperative planning, as they may directly influence implant selection and improve the accuracy of distal radius fracture fixation.

## Introduction

Distal radius fractures, representing nearly 17.5% of all adult fractures, are the most frequently encountered orthopaedic injuries and significantly affect both functional capacity and overall quality of life^1^. The volar approach to distal radius fractures offers several significant advantages that contribute to its preference in clinical practice. The volar cortex of the distal radius provides a robust anatomical structure that facilitates precise fracture reduction. Its rich vascular network supports accelerated bone healing, while the surrounding soft tissues enhance implant stability and minimize the risk of complications. In addition, the interlocking plate technology, which is particularly effective in the treatment of osteoporotic fractures, provides stable and reliable fixation. These combined benefits reinforce the effectiveness of the volar approach as the leading technique for treating distal radius fractures^2^.

 The volar surface of the distal radius is a complex anatomical region that is an essential part of any surgical procedure, especially for fixation of distal radius fractures. Accurate identification of anatomical landmarks on the volar surface is critical for successful surgical outcomes as the use of volar plate fixation has become a standard approach due to its ability to reconstruct the normal anatomical structure effectively^3^. However, the complex morphology of the distal radius, combined with individual and racial differences, often presents challenges in achieving optimal bone-implant compatibility. Implants produced in standard sizes may not be able to accommodate these variations, making it essential to consider factors such as gender, age and laterality when designing implants for these procedures. Despite the growing emphasis on individualised implant design, many procedures still rely on commercially available standard size implants that do not account for variations in individual anatomy^2^.

 Fixation with implants placed through the volar aspect is a common surgical method for restoring anatomical alignment in distal radius fractures; however, achieving a proper fit between the implant and the bone can be challenging when individual volar curvature or facet orientation does not match the standardized plate design. One clinically important result of this mismatch is inadequate stabilization of the distal volar lunate facet (VLF), which may allow displacement of the volar rim and lead to complications such as volar carpal subluxation or postoperative fragment migration^4^. These limitations have been attributed to variations in medial volar cortex morphology and lunate facet angulation, emphasizing the need for a more detailed understanding of distal radius anatomy when selecting or contouring implants. Therefore, evaluation of VLF alignment, scaphoid facet inclination (SFI), lunate facet inclination (LFI) angles, and overall volar curvature may help guide optimal fixation strategies.

 The volar projection of the lunate facet, commonly described as the teardrop, shows substantial interindividual variation. Yoneda et al. demonstrated that differences in teardrop height and inclination may reduce plate-to-bone conformity and lead to insufficient support of the volar lunate facet rim, particularly when standard pre-contoured plates are used^5^. Inadequate conformity can result in implant prominence, loss of reduction, and even flexor tendon irritation or rupture in cases where the distal edge of the plate extends beyond the watershed line^5^.

Failure to achieve proper anatomical alignment during the reduction of the lunate facet inclination (LFI) and interfacet angle (IFA) has also been associated with the development of secondary pathologies, including Kienböck’s disease^6^. This highlights the importance of evaluating potential variability in LFI and IFA measurements based on factors such as gender and laterality. Additionally, the difference in ulnar variance may predispose patients to carpal joint pathologies following distal radius fracture reduction^7^.

In the present study, anatomical details of the volar surface of the distal radius that may improve anatomical fit for volar implant surgery were analysed in three dimensions, taking into account differences in gender, age, and laterality (right-left). By integrating these multidimensional parameters, our study represents a significant advance in the understanding of anatomical factors influencing distal radius fractures and their implications for volar plating surgery. The aims of the present study were to: (a) define the volar angle of the distal radius in the intermediate and lateral columns^8^; (b) identify the volar surface width^9^; (c) assess the ulnar variance^10^; and (d) evaluate the inclinations of the scaphoid and lunate facets^11^. In addition, the study was designed to investigate the relationships between these parameters. By providing a deeper understanding of the anatomical variations in the distal radius, this study aims to contribute to the development of anatomic fit implant designs and improve surgical outcomes in the treatment of distal radius fractures.

## Materials and methods

This retrospective study evaluated computed tomography (CT) scans of the hand and wrist obtained from the institutional archive between January 2015 and May 2019. A total of 500 wrist CT scans were initially screened. Scans were included if the distal radius was intact, clearly visualized, and obtained in the neutral anatomical wrist position. Exclusion criteria were distal radius fractures, osseous defects, inflammatory or degenerative pathologies, or images acquired in pronation or supination. After applying these criteria, 103 CT scans (78 males, 25 females; 55 right and 48 left wrists) were selected for morphometric analysis. The mean age of the participants was 39.31 ± 11.05 years (range: 19–67).

The study was conducted in accordance with the Declaration of Helsinki and approved by the Acibadem University Ethics Committee (approval number: 2019-10/10). In accordance with institutional ethical policy, all CT scans were anonymized by the radiology department prior to transfer to the research team. Personal identifiers were removed, and DICOM files were reassigned with coded numerical IDs. Researchers had access only to age, sex, and imaging data. Informed consent was obtained from all subjects or their legal guardians.

Morphometric measurements were performed by two observers. Observer 1 (anatomy specialist, 9 years of experience) independently performed repeated measurements in a blinded manner for interobserver reliability assessment. Observer 2 (orthopedic surgeon, > 20 years of experience in upper extremity surgery) performed the initial measurements. A second measurement round was completed by Observer 2, and a third round by Observer 1 one month later. These data were used to evaluate inter- and intraobserver reliability.

### Construction of a 3D radius model

All CT scans were acquired in the supine position using a Somatom Force scanner (Siemens Healthineers, Erlangen, Germany) with standard protocols (100 kVp, 256 mAs), reconstructed with a standard bone mode, a slice thickness of 0.5–1.0 mm, an image matrix of 512 × 512 pixels (voxel size ≈ 0.7 × 0.7 × 0.5 mm³), and a field-of-view (FOV) between 160 and 180 mm to include the entire distal radius and ulna.

All measurements were made on the 3D radius models. The measurements were;

- The volar angle of the distal radius on the intermediate and lateral columns;
- The volar surface width;
- The differences in ulnar variance;
- Analyse the scaphoid, lunate facet, and interfacet inclinations.

### Construction of the measurements

All CT datasets were imported into the Sectra IDS7 workstation, where the distal radius and ulna were segmented to generate three-dimensional bone models. All quantitative linear and angular measurements were then performed on the 3D radius model within the Sectra environment using the built-in calibrated measurement tools. (Sectra AB, Linköping, Sweden). This platform was preferred because it provides clinically validated calibrated measurement tools and avoids potential geometric distortion that may occur after 3D surface reconstruction. Performing measurements on the raw CT data ensured that values were obtained without resampling, smoothing, or polygonal approximation of cortical surfaces.

**Fig. 1:**
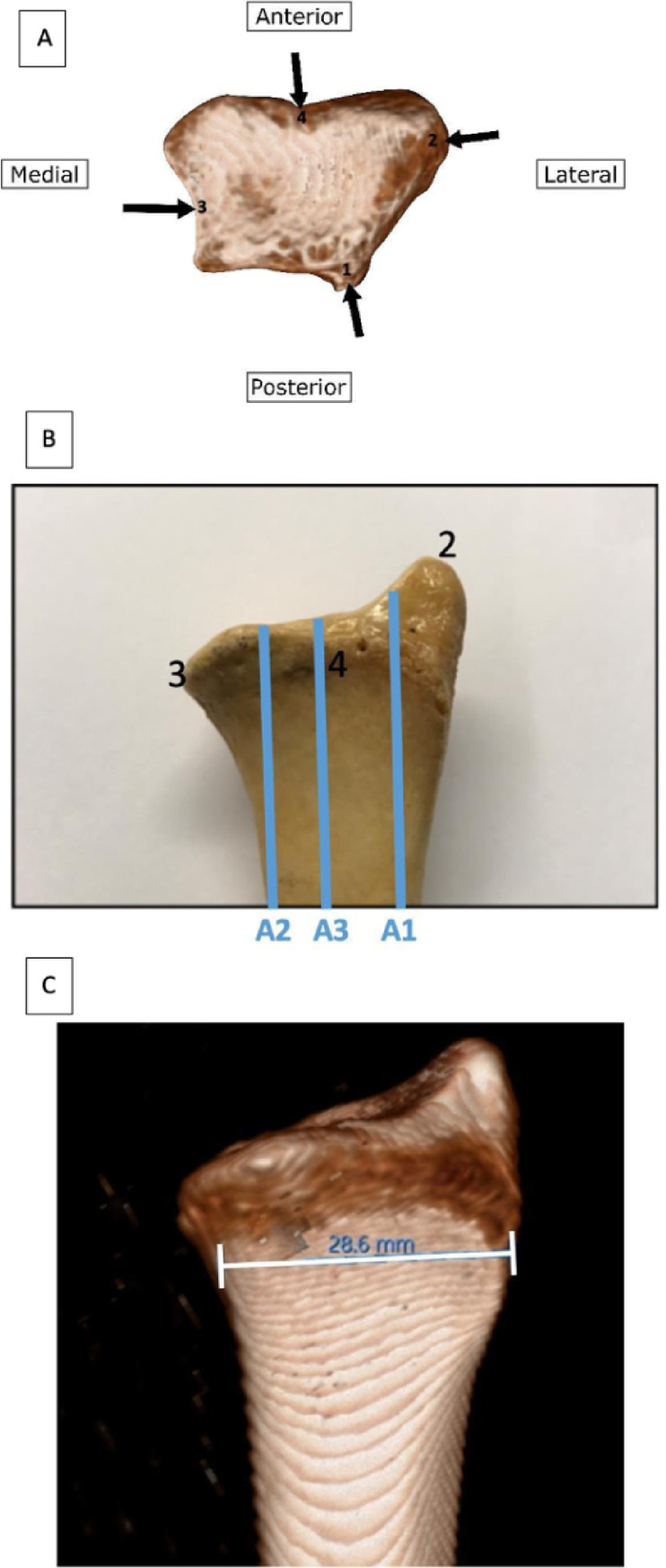
Anatomical reference points and measurement planes of the distal radius. (Fig. 1A) Reference bony landmarks: (1) the dorsal radial tubercle, (2) radial styloid process, (3) midpoint of ulnar notch (4) watershed line depth point. (Fig. 1B) Sagittal measurement planes: A1 (scaphoid facet midpoint), A2 (lunate facet midpoint), A3 (interarticular midpoint). (Fig. 1C) Distal radial width measurement (G) drawn transversely at the level of the inferior border of ulnar notch.

This workflow ensured that (1) all numeric values were derived directly from raw CT images on a clinically validated measurement platform Sectra IDS7, and (2) the reconstructed 3D models accurately illustrated the same anatomical landmarks used during measurement, allowing full reproducibility and methodological transparency. To establish a reproducible anatomical measurement, specific bony landmarks of the distal radius were identified. The primary reference points included: (1) the dorsal radial tubercul, (2) the radial styloid process, (3) the midpoint of the ulnar notch and (4) the deepest point of the watershed line. These landmarks were used to guide orientation of the distal radius in three-dimensional space and to ensure consistent alignment across all specimens (Fig. 1A).

For the assessment of volar curvature in the sagittal plane, three standardized sagittal sections were generated (Fig. 1B). Plane A1 passed through the midpoint of the scaphoid fossa, Plane A2 through the midpoint of the lunate fossa, and Plane A3 through the interfoveal region. On Plane A1, volar curvature was measured at 1 cm (A1-1) and 2 cm (A1-2) distal to the watershed line.

Distal radial width (G) was determined by drawing a transverse line at the level of the inferior border of the ulnar notch and extending it laterally to the outer radial cortex (Fig. 1C).

In all measurements, the reference planes (A1, A2, and A3) were constructed as sagittal sections passing through the specified anatomical landmarks and oriented parallel to the longitudinal axis of the radial shaft, ensuring consistent reproducibility across all CT models.

**Fig. 2:**
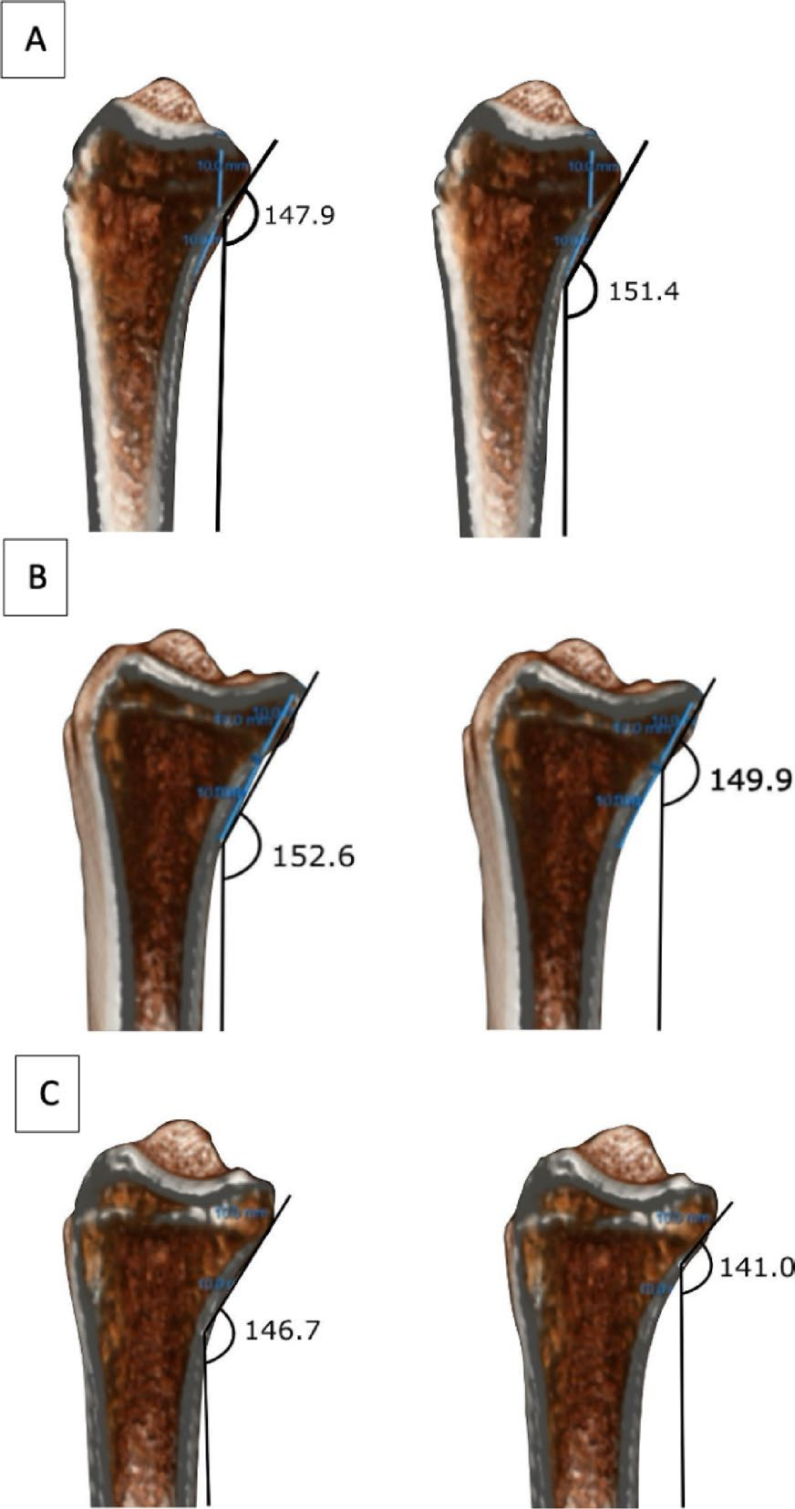
Measurement of volar curvature in standardized sagittal sections. (Fig. 2A) A1 corresponds to the sagittal plane passing through the midpoint of the scaphoid fossa, (Fig. 2B) A2 through the midpoint of the lunate fossa, and (Fig. 2C) A3 between these two planes (intermediate column). On each plane, a point 1 cm and 2 cm proximal to the distal volar rim was selected, and the volar inclination angle was defined as the angle formed between the volar cortical line and a line parallel to the radial shaft axis. The red circle marks the measurement point, and the asterisk (*) indicates the supplementary angle (180° – measured angle).

The volar angles of the distal radius were measured at A1, A2, and A3. In addition, angles at 1 cm and 2 cm proximal to the volar rim were assessed to evaluate implant fit, as in shown Fig. 2.

**Fig. 3:**
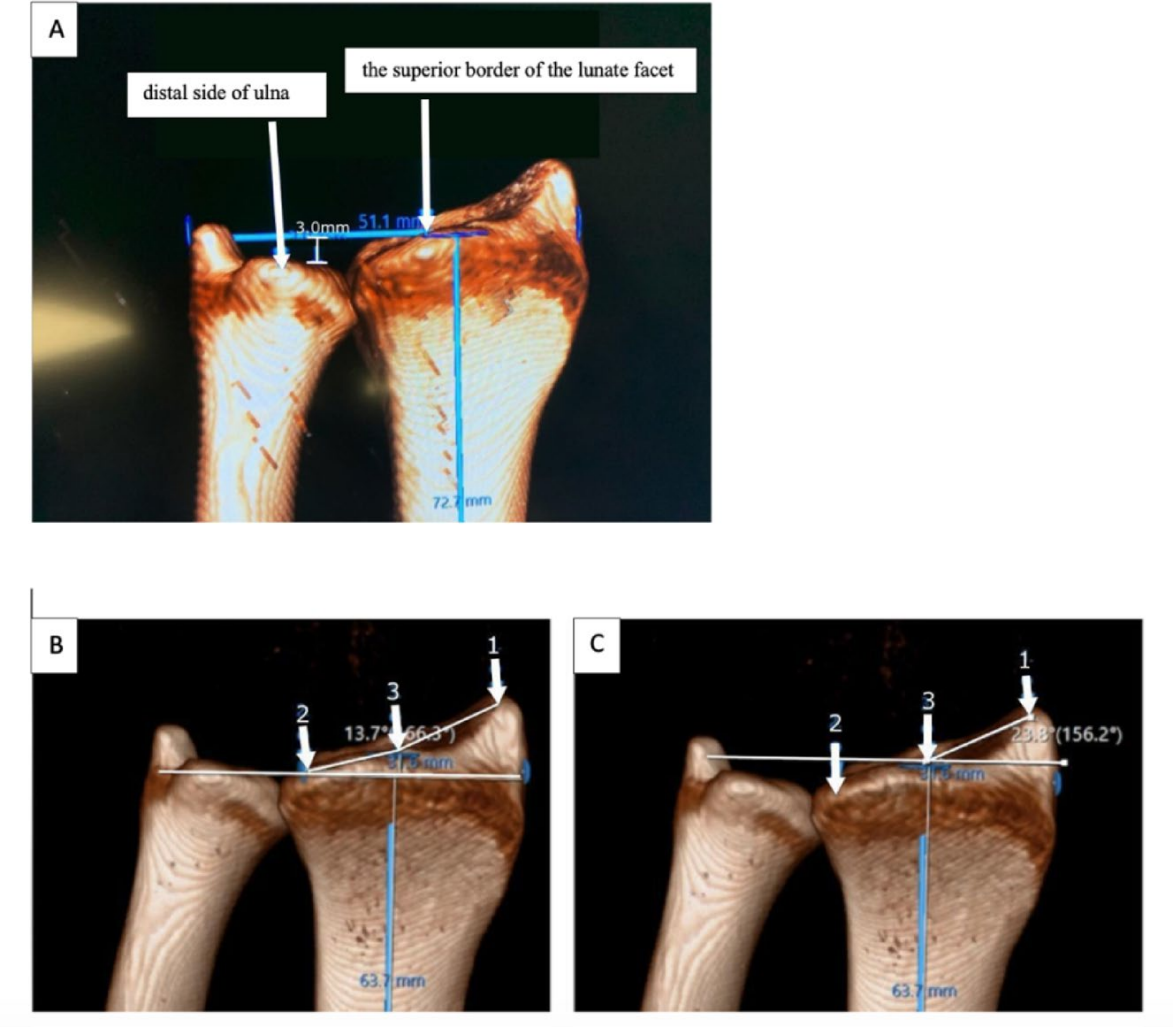
Illustration of ulnar variance measurement (− 3 mm). The ‘superior border of the lunate facet’ refers to its volar-superior contour, which represents the distal-most reference point of the radius in the volar view (Fig. 3A). Measurement of LFI, SFI, and IFA parameters. Anatomical landmark 1: the most distal aspect of the radial styloid process; Anatomical landmark 2: the most distal aspect of the lunate facet; Anatomical landmark 3: the most distal aspect of the interfossa groove. For the lunate facet inclination (LFI), a line was drawn between points 2 and 3, and the angle between this line and the horizontal reference line was measured and recorded (Fig. 3B). For the scaphoid facet inclination (SFI), a line was drawn between points 1 and 3, and the angle between this line and the horizontal reference line was measured and recorded (Fig. 3C).

Ulnar variance is defined as the difference in level between the distal end of the radius and the distal end of the ulna^10^. Ulnar variance was measured on the central coronal plane, which was defined as the slice passing through the floor of the lunate fossa. A horizontal reference line was drawn on this plane at the point where the plane intersected the lunate fossa, corresponding to the level of the fossa floor. The vertical distance between this line and the most distal point of the ulna represented the ulnar variance. The calculation of ulnar variance on three-dimensional CT images is illustrated in Fig. 3A. In this example, the distal ulna lies proximal to the reference line, indicating a negative ulnar variance of − 3 millimeters (mm) (Fig. 3A).

For the definition of the SFI, LFI, and IFA angles, several reference points were identified on the three-dimensional models, as illustrated in Fig. 3B and C. These points correspond to the anatomical landmarks described by Arik et al.^11^. In Fig. 3, point 1 represents the most distal point of the radial styloid process, point 2 represents the most distal point of the lunate facet, and point 3 corresponds to the interfossal groove, located between the scaphoid and lunate facets. These distal points (Points 2 and 3) were intentionally selected on the volar aspect of the scaphoid and lunate facets rather than on the dorsal-most articular surface. A horizontal reference line was then drawn perpendicular to the long axis of the radius. For the scaphoid facet inclination (SFI), a line was drawn between points 1 and 3, and the angle between this line and the horizontal reference line was measured and recorded. For the lunate facet inclination (LFI), a line was drawn between points 2 and 3, and the angle between this line and the horizontal reference line was measured and recorded. The interfacet angle was calculated as the difference between the two angular values: IFA = SFI – LFI.

### Data analysis

Data from the study were analysed using SPSS 26.0 software^12^. The measured parameters included volar curvature angles (A1-1, A1-2, A2-1, A2-2, A3-1, A3-2), volar surface width (G), ulnar variance (UV), and articular facet inclinations (SFI, LFI, IFA). Descriptive statistics were calculated for all measurements. Normality of the data was assessed using the Shapiro–Wilk test. Variables that met the normality assumption (A1-1, A2-1, A3-1, G, SFI, and IFA) were compared between groups using independent samples t-tests, whereas variables that deviated from normality (A1-2, A2-2, A3-2, Ulnar Variance, and LFI) were analyzed using the Mann–Whitney U test. The same normality-based approach was also applied for the right–left side comparison to maintain analytical consistency across tables.

Correlation analysis was conducted to evaluate associations among angular and morphological parameters. Pearson correlation coefficients were reported for relationships involving normally distributed variables, while Spearman rank correlation coefficients were used for relationships involving non-normally distributed variables.

The reliability and measurement reproducibility for all morphometric variables were evaluated using intraclass correlation coefficients (ICCs). For inter-observer agreement (Observer 1 vs. Observer 2), a two-way random-effects, absolute-agreement, single-measures model was used, i.e., ICC (2,1) with 95% confidence intervals (CIs). For intra-observer agreement (Observer 1, repeat vs. first), a two-way mixed-effects, absolute-agreement, single-measures model was used, i.e., ICC (3,1) with 95% CIs. According to Koo and Li (2016), CC values were interpreted as poor (< 0.50), moderate (0.50–0.75), good (0.75–0.90), or excellent (> 0.90)^13^. Single-Measures ICCs are reported; estimates from SPSS for Average-Measures are presented solely for completeness and are not used for interpretation. Because reliability was determined to be good-to-excellent for nearly all variables, the first measurements from Observer 1 were used in all primary analyses.

## Results

Statistical analyses were performed on CT images of the distal radius obtained from 103 adult individuals. Interobserver agreement was evaluated using two-way random-effects, absolute-agreement intraclass correlation coefficients ICC (2,1), and intraobserver agreement using two-way mixed-effects, absolute-agreement ICC (3,1). Overall, measurements demonstrated excellent reliability, with ICC values > 0.90 for all angular parameters (Table 1). These findings indicate that the measurement protocol was stable and reproducible across observers. Because of these high levels of reproducibility, the first observer’s initial measurements were used for all subsequent analyses.

**Table 1:** Inter- and intra-observer reliability for distal radius morphometrics.

Variable	Inter-observer ICC	95% CI	Intra-observer ICC	95% CI
A1-1	0.990	0.986–0.993	0.976	0.902–0.990
A1-2	0.992	0.979–0.996	0.987	0.972–0.993
A2-1	0.994	0.991–0.996	0.990	0.986–0.994
A2-2	0.980	0.930–0.991	0.959	0.890–0.980
A3-1	0.991	0.986–0.994	0.985	0.977–0.990
A3-2	0.993	0.961–0.997	0.980	0.971–0.987
G	0.794	0.625–0.879	0.820	0.579–0.908
Ulnar Variance	0.896	0.856–0.899	0.816	0.583–0.905
LFI	0.979	0.969–0.986	0.969	0.955–0.979
SFI	0.968	0.938–0.982	0.954	0.933–0.969
IFA	0.968	0.941–0.981	0.958	0.938–0.971

Descriptive statistics for the morphometric parameters are presented in Table 2.

**Table 2:** The mean values and the standard deviations (SD).

	Mean	SD	Minimum	Maximum	Skewness	Kurtosis
*n* = 103						
Age	39.3	11.1	19	73	0.7	0.5
A1-1◦	39.6 (140.4*)	7.0	24.0	53.8	−0.7	−0.6
A1-2◦	20.1 (159.9*)	6.0	8.0	47.4	1	2.8
A2-1◦	32.3 (147.7*)	7.9	14.8	51.5	0.3	−0.6
A2-2◦	19.2 (160.8*)	5.5	9.4	33.7	0.6	−0.4
A3-1◦	35.3 (144.7*)	7.3	17.0	55.1	−0.01	−0.1
A3-2◦	17.6 (162.4*)	5.4	5.4	36.5	1	1.5
G (mm)	26.5	2.0	19.7	31.0	−0.4	0.6
UV (mm)	−2.0	2.2	−8.2	2.9	−0.5	0.3
SFI◦	26.1	7.0	9.1	46.1	−0.1	−0.1
LFI◦	−0.1	8.4	8.4	20.5	−0.3	1.3
IFA◦	26.2	10.5	10.5	60	0.3	0.3

(*)=(180-angle)= (a round angle).

Normality was assessed using the Shapiro–Wilk test. The variables A1-1 (W = 0.99, *p* =.379), A2-1 (W = 0.98, *p* =.193), A3-1 (W = 1.00, *p* =.989), G (W = 0.99, *p* =.386), SFI (W = 0.99, *p* =.410), and IFA (W = 0.99, *p* =.308) did not significantly deviate from normality (*p* >.05) and were therefore analyzed using independent samples t-tests. In contrast, Age (W = 0.96, *p* =.004), A1-2 (W = 0.95, *p* <.001), A2-2 (W = 0.95, *p* =.001), A3-2 (W = 0.94, *p* <.001), Ulnar Variance (W = 0.95, *p* =.001), and LFI (W = 0.97, *p* =.009) demonstrated non-normal distribution and were analyzed using the Mann–Whitney U test. The volar curvature angles A1-1, A2-1, and A3-1 were significantly higher in males than females (t-test, *p* <.001), as was the G angle (t-test, *p* <.001). The IFA angle was also significantly higher in males (t-test, *p* =.035), whereas SFI did not differ significantly by sex (t-test, *p* =.593). For the non-normally distributed variables, Mann–Whitney U tests indicated that A1-2, A2-2, and A3-2 values were significantly higher in males than females (A1-2 *p* <.001; A2-2 *p* =.007; A3-2 *p* =.013). LFI was significantly higher in females (*p* =.002). Ulnar Variance and Age did not differ significantly between sexes (*p* =.132 and *p* =.208, respectively) (Table 3).

**Table 3:** The comparison between the mean values and the Gender.

M (*n* = 78), F (*n* = 25)	Mean (M)	Mean (F)	Mean Diff	t	*p*	Cohen’s d
A1-1	41.0	35.0	−5.96	−3.94	**< 0.001**	−0.906
A2-1	33.8	27.7	−6.04	−3.51	**< 0.001**	−0.808
A3-1	37.0	29.8	−7.21	−4.69	**< 0.001**	−1.078
G	27.1	24.4	−2.74	−7.19	**< 0.001**	−1.653
SFI	25.9	26.7	+ 0.86	0.54	0.593	+ 0.123
IFA	27.5	22.5	−5.02	−2.14	**0.035**	−0.492

M (*n* = 78), F (*n* = 25)	Mean (M)	Mean (F)	Mean Diff	U	*p*	Z
Age	37.9	43.4	+ 5.51	811.50	0.208	−1.259
A1-2	22.3	16.9	−5.39	429.50	**< 0.001**	−4.197
A2-2	19.9	16.8	−3.09	621.50	**0.007**	−2.720
A3-2	18.3	15.4	−2.95	653.00	**0.013**	−2.477
Ulnar Variance	−2.3	−1.5	+ 0.62	781.50	0.132	−1.507
LFI	−1.6	4.3	+ 6.20	575.00	**0.002**	−3.077

Male (M); Female (F), *p <.05, ** p < = 0.001.

**Table 4:** Comparison between the mean values of the right and left distal radius Sides.

L (*n* = 48), R (*n* = 55)	Mean (L)	Mean (*R*)	Mean Diff	t	*p*	Cohen’s d
A1-1	39.8	39.3	−0.42	0.30	0.763	+ 0.060
A2-1	31.8	32.7	+ 0.90	−0.58	0.565	−0.114
A3-1	35.5	35.0	−0.44	0.31	0.758	+ 0.061
G	26.6	26.4	−0.16	0.41	0.682	+ 0.081
SFI	25.4	26.7	+ 1.35	−0.98	0.328	−0.194
IFA	26.6	26.0	−0.62	0.30	0.765	+ 0.059

L (*n* = 48), R (*n* = 55)	Mean (L)	Mean (*R*)	Mean Diff	U	*p*	Z
Age	22.1	19.9	−2.22	1068.50	0.096	−1.663
A1-2	19.8	18.5	−1.28	1147.00	0.253	−1.144
A2-2	17.7	17.4	−0.28	1308.00	0.937	−0.079
A3-2	−2.4	−1.6	+ 0.71	1122.50	0.186	−1.322
Ulnar Variance	22.1	19.9	−2.22	1068.50	0.096	−1.663
LFI	19.8	18.5	−1.28	1147.00	0.253	−1.144

Left (L); Right (R), *p <.05, ** p < = 0.001.

No significant differences were found between the right and left sides for any parameter (*p* >.05, Table 4).

Correlation analyses demonstrated moderate to strong positive associations among the volar curvature angles measured at 1 cm distal to the articular surface. Specifically, A1-1 was moderately correlated with A2-1 (*r* =.606, *p* <.001) and A3-1 (*r* =.615, *p* <.001), while A2-1 and A3-1 demonstrated a strong correlation (*r* =.712, *p* <.001). These correlations indicate coordinated variation in volar curvature along the lateral and intermediate columns. For variables that did not meet normality assumptions, Spearman correlation analysis showed a weak positive association between age and LFI (ρ = 0.213, *p* =.031). Although statistically significant, this relationship reflects a small effect size, and therefore its clinical relevance should be interpreted cautiously. Age was not significantly associated with A1-2, A2-2, A3-2, or ulnar variance (all *p* >.05). Additionally, A1-2, A2-2, and A3-2 demonstrated moderate positive intercorrelations (ρ = 0.444–0.567, all *p* <.001), reflecting coordinated angular relationships in the distal volar curvature at 2 cm distal measurements rather than strong, clinically large effects (Table 5).

**Table 5:** Correlation matrix of distal radius morphometric Parameters.

Pearson Correlations						
Variable	A1-1	A2-1	A3-1	G	SFI	IFA
A1-1	1	**0.606** (*p* <.001)	**0.615** (*p* <.001)	**0.265** (*p* =.007)	−0.115 (*p* =.246)	−0.080 (*p* =.419)
A2-1	—	1	**0.712** (*p* <.001)	**0.270** (*p* =.006)	−0.018 (*p* =.855)	−0.043 (*p* =.669)
A3-1	—	—	1	**0.371** (*p* <.001)	−0.050 (*p* =.614)	−0.057 (*p* =.567)
G	—	—	—	1	−0.095 (*p* =.338)	−0.041 (*p* =.681)
SFI	—	—	—	—	1	**0.596** (*p* <.001)
IFA	—	—	—	—	—	1

Spearman Correlation						
Variable	Age	A1-2	A2-2	A3-2	Ulnar Variance	LFI
Age	1	−0.032 (*p* =.752)	0.013 (*p* =.894)	0.004 (*p* =.965)	0.175 (*p* =.078)	**0.213** (*p* =.031)
A1-2	—	1	**0.444** (*p* <.001)	**0.567** (*p* <.001)	**−0.250** (*p* =.011)	−0.116 (*p* =.243)
A2-2	—	—	1	**0.510** (*p* <.001)	0.073 (*p* =.461)	−0.128 (*p* =.199)
A3-2	—	—	—	1	−0.024 (*p* =.813)	−0.131 (*p* =.187)
Ulnar Variance	—	—	—	—	1	0.177 (*p* =.074)
LFI	—	—	—	—	—	1

Values represent correlation coefficients (r for Pearson, ρ for Spearman). Bold values indicate statistically significant correlations (*p* <.05). Interpretation: 0.10–0.29 = weak; 0.30–0.49 = moderate; 0.50–0.69 = moderate-strong; ≥ 0.70 = strong.

## Discussion

Distal radius fractures are common orthopedic injuries that have a significant impact on patient functionality and quality of life. The volar approach is preferred for its anatomical advantages, including the strong volar cortex for precise fracture reduction, a rich vascular supply for faster healing, and soft tissues that enhance implant stability. So understanding the anatomy of the volar surface is essential to improve surgical outcomes. The study by Ağır et al. indicates that individual variations in the volar cortical angle can affect the fit of prefixed-angle plates, underscoring the importance of accurately identifying the anatomical landmarks of the distal radius for optimal implant design and successful fracture fixation^14^.

The distal radius can be divided into three columns-the medial (ulna), intermediate (medial radius), and lateral (lateral radius)-which are significant in assessing volar curvature^15^. In our study, the distal radius was divided into three columns—intermediate, lateral, and the ridge between the scaphoid and lunate facets (A1, A2, and A3, respectively)—to accurately determine the palmar cortical angle (PCA). In our study, the mean PCA value was 140.4 ± 7 degrees for the lateral column and 147.7 ± 7.9 degrees for the medial column, indicating that the lateral column is flatter compared to the medial column. In Opperman’s study, the mean PCA value was 156.07 degrees for the lateral column and 148.25 degrees for the medial column, indicating that the medial column is flatter compared to the lateral column^8^. These inconsistencies likely reflect differences in methodology and the relatively small sample size in Opperman’s study. Our analysis, based on three-dimensional CT reconstructions of 103 radii from the Anatolian population, provides a more robust representation of true anatomical morphology. Consistent with the findings of Nalbant et al. we observed that the medial PCA—defined as the angle between the volar cortical surface of the distal radius and the longitudinal axis of the shaft—was significantly higher in men^16^.

In addition to the medial and lateral palmar cortical angles, our study also demonstrated that the curvature of the central column shows measurable gender-related variation. Specifically, the A3-2 angle was significantly higher in males than in females (18.3° vs. 15.4°, *p* =.013), indicating that morphological differences extend beyond the well-described medial and lateral aspects to include the ridge between the scaphoid and lunate facets. In the context of volar distal-radius anatomy, the gender-related difference observed in the A3-2 angle in the present study may be particularly relevant, as variation at this central ridge could influence the contour of the region overlying the flexor digitorum profundus II tendon, potentially altering its relationship to volar plate placement^3^. These findings suggest that sagittal-plane curvature is influenced by gender across all three columns of the distal radius. Moreover, the gradual decrease in curvature proximally confirms that the most pronounced volar contour is located within the distal 1–2 cm of the radius—an area known to be critical during volar plating, as improper implant placement in this region has been associated with flexor tendon irritation or rupture^17^. Although the clinical implications of these angular differences require further investigation, the consistency of these patterns across all measured regions highlights the potential relevance of incorporating individual anatomical variation into preoperative planning. Taken together, the quantitative curvature measurements provided in this study—encompassing the medial, lateral, and central columns—offer detailed three-dimensional information that may assist surgeons in anticipating variations in volar anatomy. While these measurements should not replace clinical judgment, they contribute meaningful reference data that could support more precise plate contouring, screw trajectory alignment, and implant–bone conformity. As such, they may hold value in guiding surgical decision-making, particularly in settings where patient-specific morphology differs from standard implant designs.

In the present study, the average distal volar surface width of the radius was 26.5 mm, with mean values of 24.4 mm in females and 27.1 mm in males. Nalbant et al., who also examined a similar Anatolian cohort, reported slightly higher values—27.1 mm in females and 31.6 mm in males^16^. Singh et al. reported a mean distal radius width of 28 mm in a Malaysian cohort^9^. While this value is modestly higher than ours, it remains uncertain whether this difference reflects true anatomical variation between populations or differences in imaging techniques and measurement protocols. Therefore, these cross-study comparisons should be interpreted with caution, and future multicenter investigations using standardized imaging and measurement approaches may help clarify whether population-specific differences exist. Overall, although minor numerical variations are noted across studies, current evidence does not strongly indicate clinically meaningful population-based differences in distal radius width. Thus, while individual assessment remains essential, implant width selection is unlikely to require major population-specific adjustments. Regarding volar surface width, our study found no significant gender-based difference. This contrasts with certain earlier reports but may be attributed to methodological differences between studies.

Additional parameters evaluated in our study included scaphoid facet inclination (SFI), lunate facet inclination (LFI), and interfacet angle (IFA). The mean SFI was 26.1°, compared to 33.9° reported by Arik et al. (2020) who reviewed 400 distal radius PA radiographs of Caucasians in Europe^11^. The mean LFI in our study was − 0.1°, while Arik et al. reported 13.6°. For IFA, which is the mathematical difference between SFI and LFI, the mean value in our study was 26.2°, compared to 20.3° in their findings^11^. These discrepancies may be due to differences in imaging modalities as Arik et al. used two-dimensional PA radiographs, whereas we used three-dimensional CT reconstructions, which provide more accurate measurements^11^. Gender-based comparisons in our study showed no significant difference in SFI (*p* =.216), but LFI and IFA showed significant differences (*p* =.001 and *p* =.025, respectively). Similar trends were found in the study by Arik et al.^11^. Side-by-side comparisons of right and left radius data in our study showed no statistically significant differences for SFI, LFI, or IFA (*p* >.05). Age-related changes were also assessed for other parameters in this study. LFI increased with age (*r* =.240, *p* =.015), while IFA showed a negative correlation and decreased with age (*r*=-.222, *p* =.024). These findings suggest that morphometric variations in the distal radius may complicate surgical reduction of fractures with volar implants.

In the present study, the mean ulnar variance of the 103 distal radius CT scans was − 2.01 mm, with extreme values ranging from − 8.2 mm to 2.9 mm. No significant gender difference was found (*p* =.216). To investigate whether ulnar variance changes with age, Pearson’s correlation analysis revealed a significant positive relationship between ulnar variance and age (*r* =.245, *p* =.013), indicating that ulnar variance tends to increase with advancing age. Ulnar variance is a critical morphometric parameter influencing the relationship between the distal radius and ulna and has direct implications for the surgical management of volar distal radius fractures^10^. It affects fracture healing, wrist kinematics, and anatomic alignment, and should therefore be carefully considered during implant selection to prevent mismatch^18^. Consistent with the findings of Ghalimah et al., our results demonstrate that negative ulnar variance is most frequently observed in the neutral wrist position^10^. A key strength of the present study is the integrated evaluation of ulnar variance together with SFI, LFI, IFA, and volar distal radius morphology, allowing a broader morphometric perspective than previous reports. By incorporating three-dimensional CT-based measurements and examining the relationships between multiple anatomical parameters, this study provides one of the most detailed population-specific datasets reported to date for the Anatolian population. Although numerous morphometric studies have investigated distal radius anatomy, direct comparisons across studies remain challenging due to methodological differences. Specifically, some investigations relied on plain radiographs, whereas others used cadaveric specimens, dry bone measurements, or three-dimensional CT reconstructions. Although the anatomical structures are the same, the measurement planes, definitions of reference points, and coordinate systems differ considerably across studies, which limits the ability to generate unified normative reference values.

Improper implant selection or positioning in distal radius fracture surgery may result in postoperative soft tissue complications, particularly tendon irritation or rupture^17^. Gehweiler et al. demonstrated that patient-specific three-dimensional analysis of CT analysis facilitates more accurate implant and fixation material selection, thereby improving surgical outcomes and reducing complication rates^19^. Similarly, Sato et al. reported that even when implant choice is appropriate, suboptimal placement can still lead to tendon injuries, including rupture of the flexor pollicis longus tendon^20^. Gehweiler et al. further highlighted that detailed preoperative morphological assessment assists in determining optimal screw length and fixation specifications, potentially shortening operative time^19^.

Although patient-specific implants offer an appealing concept, their routine use in acute distal radius fractures remains limited because fully customized plate fabrication requires additional planning and production time. Therefore, the main clinical relevance of our findings is the contribution to more informed preoperative planning and more appropriate implant selection rather than routine use of custom hardware. Given the substantial individual variation in distal radius morphology—such as differences in volar curvature, SFI/LFI angles, and ulnar variance—standard plates may not always provide optimal conformity. Such mismatches may, in certain cases, contribute to suboptimal reduction or soft-tissue irritation. In this context, modular plate systems, preoperative contouring guided by 3D morphometry, or the use of patient-specific surgical guides represent more feasible and currently applicable translational strategies. The morphometric reference values provided in the present study may assist these approaches by offering a more detailed anatomical framework to guide implant selection and improve surgical decision-making. As noted by Kwon et al., expanding the variety of available implants or incorporating personalized preoperative imaging strategies may enhance surgical precision and improve clinical results^21^.

Although previous studies have generally evaluated these measurements separately, assessing them together may provide a more clinically meaningful understanding of wrist biomechanics^5^. For example, increased ulnar variance has been shown to elevate ulnocarpal loading, while changes in lunate fossa inclination may alter lunate positioning on the radial articular surface^22^. Likewise, loss of volar tilt has been associated with altered carpal kinematics and increased difficulty in achieving optimal plate positioning. The clinical consequences of disrupted distal radius geometry are further supported by the findings of Lee et al., who demonstrated that malunion significantly alters midcarpal and radiocarpal kinematics during the dart-throwing motion (DTM)^23^. Their three-dimensional CT–based analysis revealed that dorsal angulation and reduced radial inclination lead to abnormal carpal alignment, altered load transmission, and diminished scaphoid and lunate mobility—changes that may contribute to ligament attenuation, synovitis, and progressive dynamic instability over time. Notably, several patients continued to experience functional limitations months after conservative treatment, indicating that even subtle deformities can adversely affect long-term wrist motion^23^.

Polat et al. reported that standard volar plate designs may not always conform to patient-specific distal radius morphology, and that anatomical mismatch can lead to implant prominence, screw penetration, and tendon irritation^22^. Moreover, Polat et al. demonstrated that several morphometric wrist parameters vary significantly with age and sex in the Turkish population, indicating that universal reference values may not reliably reflect population-specific anatomy^22^. In this context, the present study provides reproducible three-dimensional morphometric data specific to the Anatolian population, which may assist in reducing implant–bone mismatch and improving individualized preoperative planning. The clinical relevance of incorporating multiple anatomical parameters into surgical decision-making is further supported by Lee et al., who showed that distal radius malunion substantially alters midcarpal and radiocarpal kinematics during the dart-throwing motion^23^. Their three-dimensional analysis demonstrated that dorsal angulation and reduced radial inclination disrupt normal carpal alignment, modify load transmission, and diminish scaphoid and lunate mobility—mechanical alterations that may predispose patients to ligament attenuation, synovitis, and progressive instability over time. Notably, functional limitations persisted months after conservative treatment, suggesting that even subtle deviations from native anatomy can adversely affect long-term wrist mechanics^23^. Consistent with these biomechanical observations, recent evidence has further highlighted that malreduction of distal radius fractures—particularly involving volar tilt, radial inclination, or ulnar variance—can alter carpal alignment and lead to long-term functional impairment^24^. Together, these findings reinforce the value of comprehensive morphometric assessment and support the use of population-aligned three-dimensional reference data to optimize implant selection, enhance preoperative planning, and minimize postoperative complications.

In addition to the broader clinical and biomechanical considerations, several statistically significant morphometric patterns identified in the present study further highlight the importance of individualized anatomical evaluation. Volar curvature angles at both 1 cm and 2 cm distal to the articular surface (A1–A3 measurements) were consistently higher in males, demonstrating clear gender-dependent variation in sagittal-plane morphology. These differences may influence volar plate contouring, optimal plate position relative to the watershed line, and the risk of flexor tendon irritation during fixation. The strong and moderate intercorrelations observed among A1-1, A2-1, and A3-1 (*r* =.606–0.712) indicate that volar curvature varies in a coordinated manner along the lateral and intermediate columns, supporting a three-column anatomical approach rather than isolated assessment of single landmarks. Parallel patterns were observed more proximally, where A1-2, A2-2, and A3-2 showed moderate positive intercorrelations (ρ = 0.444–0.567), further emphasizing that the volar radius morphology behaves as an integrated geometric system.

Ulnar variance, although not significantly different between sexes, demonstrated a significant positive correlation with age (*r* =.245, *p* =.013), suggesting that age-related changes may alter ulnocarpal load distribution and have implications for implant choice, postoperative wrist mechanics, and long-term functional outcomes. Lunate facet inclination also showed a weak but meaningful age-related increase (ρ = 0.213, *p* =.031), and gender differences in LFI and IFA further confirm that distal radius anatomy is shaped by multiple demographic factors rather than a single morphometric determinant. Importantly, no side-to-side differences were detected for any parameter, indicating that the contralateral wrist can serve as a reliable anatomical reference when patient-specific 3D evaluation is available.

Collectively, these statistically significant findings underscore that distal radius morphology is a coordinated, multidimensional structure influenced by gender, age, and column-specific anatomical features. Integrating volar curvature, facet inclinations, and ulnar variance into preoperative planning—rather than relying on single parameters—may therefore enhance implant selection, improve plate-to-bone conformity, and reduce complications related to malreduction or implant malposition. By providing population-specific three-dimensional reference values for the Anatolian population, this study offers a robust quantitative foundation to support more precise, anatomy-informed surgical strategies in distal radius fracture management.

### Limitations of the study

This study has several limitations that should be acknowledged. First, the gender distribution within the sample was unequal, which may limit the robustness of sex-specific comparisons. Additionally, the relatively young mean age of participants restricts the generalizability of the results to older individuals, who represent the population most commonly affected by distal radius fractures. The absence of detailed anthropometric information—including hand dominance, occupational loading, or skeletal frame size—further limits our ability to assess potential external contributors to morphometric variation.

Although high inter- and intra-observer reliability supports the reproducibility of our measurement protocol, the findings should not be interpreted as conclusive evidence of population-specific anatomical distinctions. Instead, the dataset represents an initial reference model derived from a consistent 3D CT–based analytical framework. Larger, multi-center investigations including broader age groups and ethnically diverse cohorts are needed to determine whether the morphometric differences observed in this study reflect true anatomical variability or arise from sampling characteristics.

In this study, SFI and LFI were measured using the most volar distal points of the scaphoid and lunate facets, in accordance with previous work and the volar-focused anatomical perspective of the analysis. This approach was chosen to reflect how these facets contribute to volar curvature during fracture reduction and to better understand how volar implant placement may be optimized with respect to SFI and LFI. Nonetheless, this method may not fully represent the inclination of the entire articular surface along the central midline plane. Future studies could further refine this assessment by defining these landmarks on the midline articular surface, which may offer a more comprehensive understanding of facet orientation.

Despite these limitations, the three-dimensional morphometric values presented here offer clinically relevant insights. Prior research suggests that demographic and anthropometric variables may influence distal radius morphology; however, the anatomical trends identified in this study remain informative for preoperative planning, implant design, and biomechanical modeling. The present data provide a foundational reference for future comparative studies and may contribute to the development of more anatomically congruent and population-aligned surgical implants.

## Data Availability

The datasets generated and analysed during the current study are not publicly available due to the nature of the data (reconstructed radiological CT images obtained from the hospital archive), but are available from the corresponding author on reasonable request.
